# Intra-Operative Detection of a Left-Sided Non-Recurrent Laryngeal Nerve during Vagus Nerve Stimulator Implantation

**DOI:** 10.3390/medicina56100489

**Published:** 2020-09-23

**Authors:** Jason John Labuschagne, Niels Hammer

**Affiliations:** 1 Netcare Unitas Hospital, Centurion 0140, South Africa; 2 Department of Neurosurgery, University of Witwatersrand, Johannesburg 2193, South Africa; 3 Department of Pediatric Neurosurgery, Nelson Mandela Children’s Hospital, Johannesburg 2193, South Africa; 4 Department of Macroscopic and Clinical Anatomy, Medical University of Graz, 8010 Graz, Austria; 5 Department of Trauma, Orthopedic and Plastic Surgery, University Hospital of Leipzig, 04109 Leipzig, Germany; 6 Fraunhofer Institute for Machine Tools and Forming Technology, 01187 Dresden, Germany

**Keywords:** nerve stimulation, non-recurrent laryngeal nerve, seizure treatment, vagus nerve, vagal trunk, variational anatomy

## Abstract

Left sided non-recurrent laryngeal nerves (NRLN) are very rarely observed during surgery in the head and neck region. Arising directly from the cervical aspect of the vagus nerve, the NRLN lies in a vulnerable position distant from its normal location. NRLNs are normally associated with embryological branchial arch aberrations and subsequent vascular anomalies. The anomalous course of the NRLN makes it more susceptible to injury during surgery in the neck region. Knowledge of this anatomical variant will reduce the potential for injury and resultant vocal cord paralysis. During microsurgical dissection of the carotid sheath for the implantation of a vagus nerve stimulator in a 19-year-old female patient with refractory epilepsy, a moderate-sized branch of the main vagus nerve trunk was identified postero-medially within the carotid sheath. Intra-operative stimulation of this nerve resulted in a compound muscle evoked potential from the left vocal cord. Thus, this branch was confirmed to be a left-sided NRLN. The patient had no associated vascular anomalies. This is first reported case of a left-sided NRLN found during VNS insertion. Awareness of the possibility of an NRLN is imperative to prevent iatrogenic injury. A medial location of the vagus nerve within the carotid sheath should alert the surgeon to the possible presence of an NRLN. The absence of fourth branchial arch remnant anomalies is not a guarantee as to the absence of a left-sided NRLN. The addition of intra-operative nerve monitoring for vagus nerve stimulator (VNS) implantation procedures should be strongly considered to help avoid iatrogenic injury.

## 1. Introduction

Left sided non-recurrent laryngeal nerves (NRLN) are very rarely observed during surgery in the head and neck region. In “non-anomalous” cases, the right recurrent laryngeal nerve (RLN) arises from the right vagus nerve anterior to the first part of the subclavian artery, it then hooks around the right subclavian artery and ascends towards the tracheo-oesophageal groove before entering the larynx [1]. On the left side, the RLN is given off by vagus at the inferior border of the arch of the aorta, it then passes inferior to the arch of the aorta, immediately lateral to the ligamentum arteriosum, and ascends in the tracheo-oesophageal groove to the larynx [1]. Non-recurrent laryngeal nerves arise directly from the cervical aspect of the vagus nerve to enter the larynx directly without first descending to the thoracic level. NRLNs have been reported in only 0.3–0.8% [2] of the population on the right side, and they are extremely rare on the left side, with a reported incidence of less than 0.004% [2]. Indeed, to our knowledge, only four cases of left-sided NRLNs have been reported [3].

The anomalous course of the NRLN makes it more susceptible to injury during surgery in the neck region. Knowledge of both the normal anatomy and anatomical variants of the recurrent laryngeal nerve should serve to reduce iatrogenic injury to the nerve. We report a case of a left NRLN discovered during vagus nerve stimulator implantation (VNS).

## 2. Case Report

A 19-year-old female patient with drug-resistant epilepsy, who was not considered a suitable candidate for resective epilepsy surgery, was offered vagal nerve stimulation for seizure control.

Surgery was performed under general anaesthesia after the administration of intravenous antibiotics and under strict aseptic protocol. The patient was placed supine with the head in the midline and a slightly extended position. A horizontal neck incision was made at the level of the mid larynx, halfway between the clavicle and mastoid process. The sternocleidomastoid muscle was dissected along its anterior aspect and retracted posteriorly. A combination of sharp and blunt dissection was used to expose a 4-cm long segment of the carotid sheath. The fibrous connective tissue of the sheath was opened with atraumatic forceps by splitting the fibres of the sheath medially and laterally to reveal the contents of the carotid sheath. Further dissection was performed under microscopic visualisation. (Zeiss, Kinevo 900, Carl Zeiss AG, Oberkochen, Germany).

**Figure 1:**
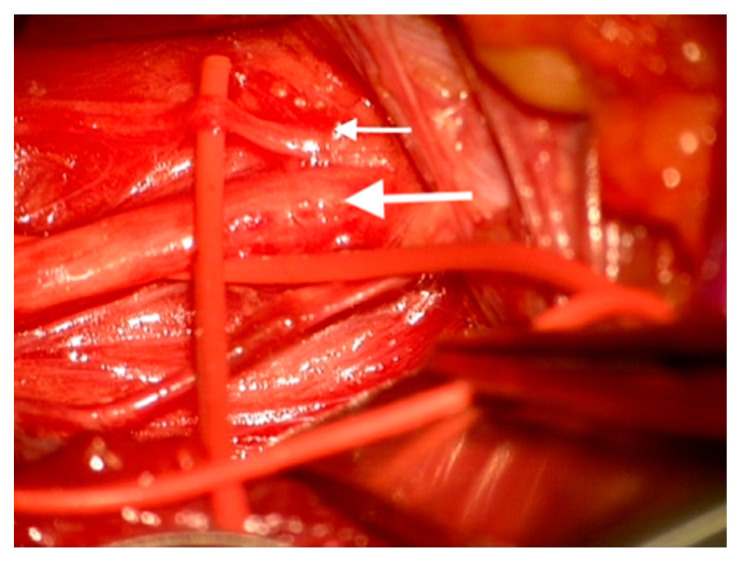
Intra-operative images showing an aberrant branch (small white arrow) of the left vagus nerve (large white arrow) in the cervical sheath identified as a non-recurrent laryngeal nerve.

During dissection of the carotid sheath, a moderate-sized branch of the main vagus nerve trunk was identified postero-medially within the carotid sheath (Figure 1). Vessel loops were placed around the proximal and distal ends of the main vagal trunk and around the proximal portion of the medial branch. Intra-operative stimulation (0.3 mA monopolar stimulation) was delivered by a commercially available neurostimulator (Medtronic NIM^®^ Eclipse, Minneapolis, MN, USA) to the vagus trunk and to the medial branch. The vocal cord responses were recorded by a commercially available EMG embedded endotracheal tube device used to detect vocal cord responses (Medtronic NIM^®^ Flex, EMG Tube, Minneapolis, MN, USA). Stimulation of the common trunk and medial branch both elicited a compound muscle evoked potential from the left vocal cord. A similar latency period of 6 ms was detected following stimulation of both the trunk and the medial branch; however, the amplitude of the compound muscle potential was approximately half when the vagal trunk was stimulated as opposed to the medial branch stimulation.

**Figure 2:**
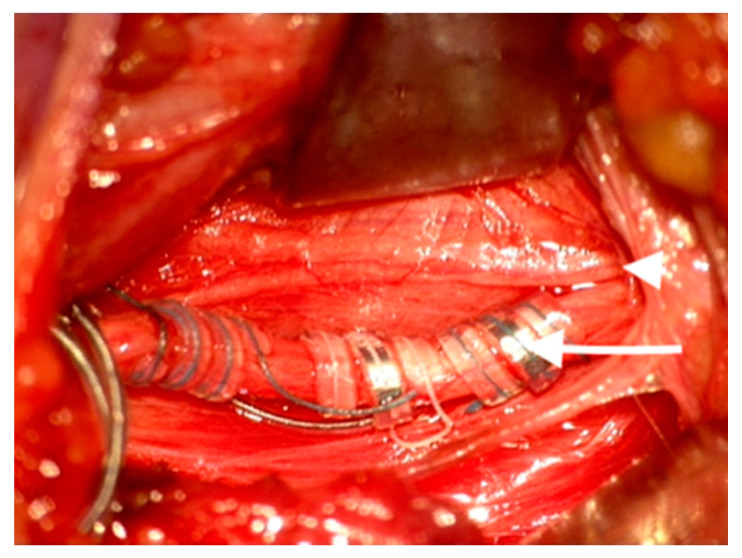
Vagus nerve stimulator (VNS) electrode placed around the main vagal trunk (white arrow) with the aberrant branch displaced medially at the branching point (white arrowhead) on the left side.

The VNS coils of the VNS Stimulator (AspireSR, model 106, Cyberonics Inc. Houston, TX, USA) were placed around the main vagal trunk, away from the branching point. (Figure 2). 

The lead wire was passed subcutaneously to an infraclavicular subcutaneous pocket. Then, the lead was attached to the implantable pulse generator. There were no cardiac responses to intra-operative test stimulation. The wound was closed in anatomical layers. The patient had no intra-operative nor post-operative complications. A routine chest X-ray was performed as part of the pre-operative workup, and this did not demonstrate evidence of situs invertus or a right-sided aortic arch. Additional investigations for vascular anomalies were not performed.

The patient was seen for regular follow-up visits. During follow up, a structured questionnaire designed to elicit any laryngopharyngeal symptoms was administered. The patient presented no evidence of vagal nerve dysfunction. At six-month follow up, she had responded well to the VNS, with a reduction in seizure frequency and severity.

## 3. Discussion

To our knowledge, this is the first reported case of an NRLN in the setting of VNS implantation and one of the few cases in which the course of the NRLN is described both within the carotid sheath and once exiting the sheath. According to our knowledge, it is also the first reported case to demonstrate the benefit of standard intra-operative nerve monitoring (IONM) in the setting of VNS implantation in delineating anatomical variations in this region and the potential of IONM to assist in avoiding iatrogenic injury.

Embryologically, the recurrent laryngeal nerves (RLNs), also frequently referred to as the “inferior laryngeal nerves”, are derived from the VI branchial arch and have a horizontal course. Subsequently, the V and distal portion of the VI branchial arches regress bilaterally, and the RLNs remain anchored to structures that develop from the IV branchial arch: the aortic arch on the left and the subclavian artery on the right [4,5]. As the heart descends and the neck elongates, the nerves are pulled into an intrathoracic position, and therefore, they need to assume a recurrent course. The recurrent laryngeal nerves (RLNs) run a different course on each side. On the right, they arise from the right vagus nerve anterior to the first part of the subclavian artery. Then, the right RLN hooks around the right subclavian artery and ascends towards the tracheo-oesophageal groove before entering the larynx [1]. On the left side, the RLN is given off by vagus at the inferior border of the arch of the aorta. The left RLN passes inferior to the arch of the aorta, immediately lateral to the ligamentum arteriosum, and ascends in the tracheo-oesophageal groove to the larynx [1].

The NRLN is a rare anomaly in which the NRLN branches off the cervical vagal trunk to enter the larynx directly from within the carotid sheath, without first descending to the thoracic level. It has been reported in only 0.3–0.8% [2] of the population on the right side, and it is extremely rare on the left side, with a reported incidence of less than 0.004% [2]. Indeed, to our knowledge, only four cases of left-sided NRLNs have been reported [3].

Three types of NRLN have previously been distinguished [6]: type 1 NRLNs arise directly from the vagus and run together with the vessels of the superior thyroid peduncle; type 2a NRLNs follow a transverse path parallel to and over the trunk of the inferior thyroid artery; and type 2b NRLNs follow a transverse path parallel to and under the trunk or between the branches of the inferior thyroid artery. The case presented here is an example of a type 1 NRLN.

Most descriptions of the NRLN describe the path of the nerve once it exits the carotid sheath, as this is of most relevance to thyroid and parathyroid surgery. Very little is described regarding NRLN anatomy within the carotid sheath. Gurleyik [7] described a case of a right-sided NRLN discovered during a total thyroidectomy in which they followed the NRLN back into the carotid sheath and found that the length of the nerve from its origin, the trunk of the vagus nerve, to the laryngeal entry point was 4 cm. In this case report of Gurleyik [7], the trunk of the vagus nerve ran parallel and antero-medially within the carotid sheath. The association between the vagus nerve being located medially, within the carotid sheath, to the common carotid and the presence of a NRLN is emphasised by Toniato et al. [6], who describe this medial location of the vagal trunk as a “pilot light” to the possible presence of an NRLN. However, this medial location is not an absolute, as Coady et al. [8] described a case of a left NRLN in a patient undergoing a carotid endarterectomy in which the vagus nerve was anterolateral to the carotid artery and the NRLN crossed anterior to the carotid to reach the larynx. In the present case, the vagus trunk was in a postero-medial location to the common carotid.

Generally speaking, the non-recurrence of the right laryngeal nerve results from a vascular anomaly during embryonic development of the aortic arches, with the resultant absence of the innominate artery and the formation of an aberrant right subclavian artery, the so-called arteria lusoria, which arises directly from the aorta left of the midline and crosses the oesophagus. For non-recurrence of the left-sided laryngeal nerve, a triad of fourth branchial arch remnant anomalies are required: a right-sided aortic arch with an associated situs inversus; a right-sided ligamentum arteriosum, and a left subclavian artery with a lusoria course [9]. However, up to 11% of reported right-sided NRLNs are not associated with a subclavian artery abnormality [10]. To our knowledge, only one case [8] prior to the currently presented case has been reported of a left NRLN without an associated vascular anomaly. The current case had no evidence of fourth branchial arch anomalies. The embryological pathogenesis of an NRLN with an associated vascular anomaly is understood, but the presence of the variant nerve without the accompanying vascular anomaly “remains a mystery” [10]. Some authors have challenged the viability of an NRLN without a branchial arch anomaly [9,11,12] and have noted that “the origin of the non-recurrent nerve was never confirmed as the vagus nerve” [12]. Raffaelli et al. [9] posit that an NRLN in the patients without vascular anomaly is in fact a “false NRLN”. This “false NRLN” is thought to be an anastomotic nerve between the sympathetic chain and the RLN that has a similar diameter and size as an NRLN [13]. In their manuscript, Tateda et al. [3] reported a case of NRLN without a vascular anomaly, in whom vocal cord mobility was proven by intra-operative nerve stimulation of the NRLN. If in this case it was a “false NRLN”, it would imply that the sympathetic trunk is involved in vocal cord function, which has never been shown to be the case [3]. Likewise, in the case presented here, IONM confirmed a compound action potential from the left vocal cord implying a “true” NRLN.

As far as we are aware, this is the first reported case of an NRLN in the setting of VNS therapy. Despite the widespread use of intra-operative nerve monitoring (IONM) in head and neck endocrine surgery [14,15], IONM is not routine practice for VNS insertion, with few surgical units [16] describing its use to minimise voice-related side effects. Interestingly, Dolezel et al. [11] reported that the use of IONM in thyroid surgery increased the prevalence of NRLN detection, yet it decreased the incidence of post-operative palsy. Our unit uses IONM as standard practice, and in the current case, its availability was invaluable in confirming the presence of the NRLN.

## 4. Conclusions

Awareness of the possibility of an NRLN within the carotid sheath during VNS implantation or any other surgery involving dissection of the carotid sheath is imperative to prevent iatrogenic injury to the vagus nerve and its branches. A medial location of the vagus nerve within the carotid sheath should alert the surgeon to the possible presence of an NRLN. The absence of fourth branchial arch remnant anomalies is not a guarantee as to the absence of a left-sided NRLN. The addition of IONM for VNS implantation procedures should be strongly considered to help avoid iatrogenic injury.
